# CircNOL10 suppresses breast cancer progression by sponging miR-767-5p to regulate SOCS2/JAK/STAT signaling

**DOI:** 10.1186/s12929-020-00697-0

**Published:** 2021-01-04

**Authors:** Fang Wang, Xiaochun Wang, Jingruo Li, Pengwei Lv, Mingli Han, Lin Li, Zhuo Chen, Lingling Dong, Nan Wang, Yuanting Gu

**Affiliations:** 1 Department of Breast Surgery, The First Affiliated Hospital of Zhengzhou University, No.1 Jianshe East Road, Erqi, Zhengzhou, 450000 China; 2 Department of Breast Surgery, Affiliated Hospital of Hebei University, Baoding, 071000 China

**Keywords:** Breast cancer, Circular RNA, circNOL10, miR-767-5p, SOCS2, JAK/STAT signaling

## Abstract

**Background:**

Circular RNAs (circRNAs) have caught increasing attentions and interests for their important involvement in cancer initiation and progression. This study aims to investigate the biological functions of circNOL10 and its potential molecular mechanisms in breast cancer (BC).

**Materials and methods:**

qRT-PCR and western blot assays were performed to measure the expression of related genes. CCK-8, colony formation, flow cytomerty and transwell assays were used to assess cell proliferation, cell cycle, migration and invasion. RNA pull-down, luciferase reporter and RIP assays were applied to address the potential regulatory mechanism of circNOL10.

**Results:**

CircNOL10 was down-regulated in BC tissues and cells. Low expression of circNOL10 was associated with larger tumor size, advanced TNM stage, lymph node metastasis and unfavorable prognosis. Overexpression of circNOL10 inhibited cell proliferation, migration, invasion and EMT in vitro and slowed xenograft tumor growth in vivo. Mechanistically, circNOL10 could act as a molecular sponge for miR-767-5p, leading to the up-regulation of suppressors of cytokine signaling 2 (SOCS2) and inactivation of JAK2/STAT5 pathway. Moreover, circNOL10-mediated suppression of malignant phenotypes was attenuated by miR-767-5p. Similar to circNOL10, enforced expression of SOCS2 also resulted in the suppression of cell proliferation and metastasis. Furthermore, knockdown of SOCS2 reversed the tumor-suppressive effect induced by circNOL10.

**Conclusions:**

CircNOL10 repressed BC development via inactivation of JAK2/STAT5 signaling by regulating miR-767-5p/SOCS2 axis. Our findings offer the possibility of exploiting circNOL10 as a therapeutic and prognostic target for BC patients.

## Background

Breast cancer (BC) is a common malignant tumor among females [1]. It is estimated that BC accounts for 30% of new cancer diagnoses in women and 14% of cancer-related death in females [2]. Despite some improvements in therapeutic strategy, such as medication, radiation therapy, and surgery, the survival rate of BC patients at an advanced stage is still low [3]. Therefore, it is imperative to explore the molecular mechanism of BC for identifying more effective biomarkers and therapeutic targets.

Circular RNAs (CircRNAs), a family of covalently closed loop structure RNAs without 5′-cap and 3′-poly A tail, have been identified to play vital roles in human cancers [4, 5]. As a kind of conserved and stabilized non-coding RNAs, circRNAs always display a species-, tissue- and cell-specific expression pattern [6]. In recent years, it has been found that circRNAs can participate in diverse biological processes by serving as miRNA sponges, binding RNA binding proteins (RBPs), working as a transcription factor, and translating proteins [7]. Increasing circRNAs are reported as oncogenic drivers or tumor suppressors in various human malignancies, implying a great promise of circRNAs as predictive biomarkers and therapeutic targets in cancer [8]. According to a recent document, a large number of circRNAs are discovered to be abnormally expressed in BC, and are involved in carcinogenesis, metastasis or chemoresistance [9]. For example, circUBXN7 suppressed cell proliferation and invasion in BC via acting as a competitive endogenous RNA (ceRNA) of miR-1247-3p to elevate B4GALT3 expression [10]. Circ-DNMT1 contributed to BC progression via activating autophagy by directly binding to p53 and Auf1 [11]. CircNOL10 (formed by circularization of 6–12 exons of Pre-NOL10; circBase ID: hsa_circ_0000977) is located at chr2:10784445-10808849 with a length of 562 bp. A previous research revealed that circNOL10expression was reduced in lung cancer tissues and cells, and circNOL10 overexpression repressed cell proliferation and induced apoptosis through transcriptional regulation of the HN polypeptide family by SCML1 [12]. Through analyzing the GEO database (GSE101123), circNOL10 was found to be significantly decreased in BC tissues. Thus, circNOL10 was selected for further function and mechanism analysis in BC progression.

MicroRNAs (miRNAs), a class of small RNAs (~ 22 nucleotides (nt)) with no translation capacity, are able to suppress gene expression via decreasing messager RNAs (mRNAs) translation or mediating mRNAs degradation [13]. MiRNAs have been widely elucidated as important regulators in cancer initiation and development [14]. To our knowledge, circRNAs could regulate gene expression through serving as “miRNA sponges” to sequester miRNAs from binding to their target mRNAs [15]. Herein, we focus on the biological roles of circNOL10 in BC and identify whether circNOL10 could affect cell malignant phenotypes through ceRNA regulating cascades “circRNA-miRNA-mRNA”.

In this study, we confirmed the down-regulation of circNOL10 in breast cancer tissues and cells. Moreover, circNOL10 inhibited cell proliferation, migration, invasion and epithelial-mesenchymal transition (EMT) by sponging miR-767-5p and regulating SOCS2/JAK/STAT signaling. This study provides new insights into the regulatory mechanisms of circNOL10 and highlights a potential molecular target in BC.

## Materials and methods

### Tissues collection

Sixty tumor tissue samples and adjacent non-cancerous specimens were obtained from BC patients who were undergoing surgery at the First Affiliated Hospital of Zhengzhou University. All patients were pathologically diagnosed as BC by two experienced pathologists. No patients suffered from other malignancy and received preoperative chemotherapy or radiotherapy. All samples were instantly frozen at − 80 °C until further analysis. Written informed consents were provided by all patients.

### Quantitative real-time polymerase chain reaction (qRT-PCR)

Total RNA in BC tissue samples and cells was extracted using TriQuick Reagent (Solarbio, Beijing, China). The random primers (TaKaRa, Dalian, China) were used for reverse transcription of circNOL10 and SOCS2, and miRNA 1st Strand cDNA Synthesis Kit (Vazyme, Nanjing, China) was utilized for reverse transcription of miR-767-5p. The quantitative PCR was performed using SYBR Premix Ex Taq II (TaKaRa) on an ABI 7500 Real-Time PCR System (Applied Biosystems, Carlsbad, CA, USA). The level of miR-767-5p was normalized by small nuclear RNA U6, while circNOL10 and SOCS2 were analyzed with glyceraldehyde 3-phosphate dehydrogenase (GAPDH) as a control. The relative RNA expression was calculated with the method of 2^−ΔΔCt^. The primers listed in Additional file 1: Table S1.

### Cell culture

Six human BC cell lines (MCF-7, MDA-MB-231, MDA-MB-468, SKBR-3, BT-549, and BT-474) and a normal breast epithelial cell line MCF-10A were purchased from Shanghai Institute for Biological Sciences (Shanghai, China). The cells were cultured in RPMI1640 medium (Invitrogen; Thermo Fisher Scientific, Inc., Waltham, MA, USA) containing 10% fetal bovine serum (Invitrogen; Thermo Fisher Scientific, Inc.) at a 37 °C incubator with 5% CO_2_. Cell line authentication was performed by short tandem repeat (STR) fragment analysis to avoid the risk of cross-contamination. All cell lines were free of mycoplasma contamination.

### Cell transfection

Small interfering RNAs (siRNAs) targeting the junction sequence of circNOL10 (si-circ #1, si-circ #2, and si-circ #3), control non-specific oligonucleotides (si-NC), siRNA targeting SOCS2 (si-SOCS2), miR-767-5p mimics (miR-767-5p) and scrambled control (miR-NC) were obtained from GenePharma (Shanghai, China). The fragments of circNOL10 and SOCS2 were amplified and then inserted into pLCDH-ciR vector (Geneseed, Guangzhou, China) or pcDNA3.1 vector (Geneseed, Guangzhou, China) to construct circNOL10- or SOCS2-overexpressing plasmid, namely circNOL10 and SOCS2. Synthetic oligonucleotides or vectors were transfected into BC cells by using Lipofectamine 3000 (Invitrogen, Carlsbad, CA, USA) according to the manufacturer’s manual.

### Circular structure confirmation

To confirm the circular characteristic of circNOL10, the circular and linear transcripts of NOL10 were amplified by divergent and convergent primers in both complementary DNA (cDNA) and genomic DNA (gDNA) from BC cells. Subsequently, agarose gel was used to separate the PCR products. Theoretically, the circular transcript of NOL10 is only amplified by divergent primers in cDNA rather than gDNA. In addition, Sanger sequencing was carried out to confirm the sequence of circNOL10.

### RNase R and Actinomycin D treatment

For RNase R treatment, 3 μg of total RNA extracted from BC cells were incubated for 20 min at 37 °C with or without 3U/μg RNase R (Epicenter Technologies, Madison, WI, USA). For Actinomycin D treatment, 2 mg/ml Actinomycin D (Sigma-Aldrich, St. Louis, MO, USA) was added to culture medium. Following treatment with RNase R or Actinomycin D, qRT-PCR was applied to measure the expression levels of circNOL10 and NOL10 mRNA.

### Cell counting kit-8 (CCK-8) assay

CCK-8 assay was performed to evaluate BC cell viability. Cells were inoculated in 96-well plates and cultured in suitable condition for 24 h, 48 h, 72 h, and 96 h. Then, 10 μl CCK-8 solution (Dojindo, Kumamoto, Japan) was dispensed to each well. After incubation at 37 °C for 2 h, the absorbance at 450 nm was measured using a microplate reader (BioTek, Winooski, VT, USA).

### Colony formation assay

Approximately 500 transfected BC cells were added into 6-well plates and incubated at 37 °C for 2 weeks to form colonies. The medium was refreshed every 3 days. Finally, the colonies were fixed with 4% paraformaldehyde and stained with 0.1% crystal violet. The number of colonies with > 50 cells was counted.

### Cell cycle assay

After incubation in the standard condition for 48 h, BC cells were harvested and fixed in pre-cold 70% ethanol at 4 °C overnight. Then, cells were centrifuged, washed with pre-cooled PBS and stained with a mixture of 50 μg/ml propidium iodide (PI) and 100 μg/ml RNase A for 30 min. A FACSCalibur flow cytometry (BD Biosciences, San Jose, CA, USA) was used to determine cell cycle distribution.

### Transwell assay

Cell migration and invasion were detected by using 24-well 8 µm pore size Transwell chambers (Corning, Tewksbury, MA, USA) coated with or without Matrigel matrix (BD Biosciences, Franklin Lakes, NJ, USA). The lower chamber was supplemented with 500 μl of RPMI1640 medium containing 10% FBS, while 200 μl cell suspension was added into the upper chamber. After 24-h of incubation, cells on the bottom surfaces of top chamber were fixed with 4% methanol and stained with 0.1% crystal violet. A light microscope (Olympus, Japan) was used to count the cells in five randomly selected fields.

### Bioinformatics analysis

The interaction between circNOL10 and miR-767-5p was predicted by CircInteractome (https://omictools.com/circinteractome-tool) and CircBank (http://www.circbank.cn/) online databases. TargetScan (http://www.targetscan.org/vert_71/), miRDB (http://mirdb.org/), and DIANA-microT-CDS (http://diana.imis.athena-innovation.gr/DianaTools/index.php?r=microT_CDS/index) were applied to identify the candidate targets of miR-767-5p.

### Isolation of cytoplasmic and nuclear RNA

NE-PER Nuclear and Cytoplasmic Extraction Reagents (Thermo Scientific, Inc., Waltham, MA, USA) was used to perform the RNA isolation of nuclear and cytoplasmic fractions in BC cells following the manufacturer’s protocol. qRT-PCR was used to detect the relative level of circNOL10 in different cellular fractions, with GAPDH and U6 as cytoplasmic and nuclear control.

### Biotinylated RNA pull-down assay

The pull-down experiments were conducted in BC cells by using biotinylated circNOL10 probe or biotinylated miR-767-5p as previously described [16]. qRT-PCR analysis was used to detect the abundance of target miRNA or mRNA.

### Dual-luciferase reporter assay

The wild type sequences of circNOL10 or SOCS2-3′UTR containing the miR-767-5p binding sites were amplified and inserted into dual luciferase reporter vector pmirGLO (Promega, Madison, WI, USA). The mutant version of circNOL10 or SOCS2-3′UTR in which the miR-767-5p binding sites were replaced was cloned into the same luciferase reporter. Then, BT-549 and MDA-MB-231 cells were co-transfected with the constructed luciferase reporter, pRL-TK Renilla luciferase reporter and miR-NC or miR-767-5p by using Lipofectamine 3000 (Invitrogen). At 48 h after transfection, Dual-Lucy assay kit (Solarbio, Beijing, China) was used to examine the luciferase activity. The Renilla activity was used as the internal reference.

### RNA immunoprecipitation (RIP) assay

Magna RNA immunoprecipitation kit (Millipore, Billerica, MA, USA) was used to analyze the binding specificity between circNOL10 and miR-767-5p. Briefly, miR-NC- or miR-767-5p-transfected BT-549 and MDA-MB-231 cells were collected, washed with phosphate buffer solution (PBS) and lysed in RIP lysis buffer containing protease and ribonuclease inhibitors. Subsequently, 100 μl of cell lysates were cultured with RIP buffer containing magnetic beads coupled with human anti-Ago2 antibody or control IgG. Following elution, qRT-PCR was performed to detect the enrichment of circNOL10 in the RNA precipitates.

### Western blot assay

Total protein was extracted from cells or tissues by using RIPA buffer (Solarbio, Beijing, China). The concentration of protein samples was assessed by a Pierce BCA protein assay kit (Thermo Scientific, Rockford, IL, USA). Equal amount of 30 μg protein samples were separated by sodium dodecyl sulfonate-polyacrylamide gel electrophoresis (SDS-PAGE), and transferred onto a polyvinylidene fluoride (PVDF) membrane. Then, the membrane was blocked in the non-fat milk for 2 h at room temperature and probed with primary antibodies against E-cadherin, N-cadherin, Vimentin, SOCS2, JAK2, p-JAK2, STAT5, p-STAT5 and GAPDH (Abcam, Cambridge, MA, USA) at 4 °C overnight. After incubation with goat anti-rabbit IgG-HRP secondary antibody (Abcam) at 37 °C for 2 h, the protein signaling was visualized via eyoECL plus kit (Beyotime, Shanghai, China). The original data for western blots were presented in Additional file 2.

### Tumor xenograft model

The 4-week-old female BALB/c nude mice (Shanghai SLAC Laboratory Animal Company, Shanghai, China) were randomly divided into 2 groups, with 6 mice in each group. For xenograft experiments, BT-549 cells (4 × 10^6^) infected with lenti-circNOL10 or lenti-vector were implanted into the right flanks of nude mice. The tumor volumes were determined every 4 days in accordance with the formula: volume (mm^3^) = width^2^ × length/2. After 27 days, mice were killed and tumor tissues were removed for further research.

### Immunohistochemistry (IHC)

The tumor tissues from nude mice were fixed with 4% paraformaldehyde, embedded in paraffin, and cut into 5-μm-thick sections. The slides were subjected to antigen retrieval and incubated with primary antibody against Ki-67 (Cell Signaling Technology, Beverly, MA, USA) overnight at 4 °C, followed by treated with HRP-conjugated secondary antibody for 1 h at room temperature. Then, the tissue sections were stained with diaminobenzidine and haematoxylin. Images were captured by using a BX51 microscope (Olympus, Tokyo, Japan).

### Statistical analysis

All data were exhibited as the means ± standard deviation (S.D.) from three independent experiments using GraphPad Prism 7 (GraphPad Inc., San Diego, CA, USA). The comparisons between two groups were analyzed by two-tailed Student’s *t*-test. One-way analysis of variance (ANOVA) followed by Dunnett’s test was used to compare the difference among three or more groups. The association of circNOL10 expression with clinicopathological features was examined by χ^2^ test. Pearson’s correlation coefficients were applied to determine the correlation between circNOL10, miR-767-5p and SOCS2 in BC tissues. The median expression of circNOL10 was used as the cut-off value to divide patients into high and low expression groups. Overall survival curves were plotted by Kaplan–Meier method and analyzed using the log-rank test. All statistical tests were two-sided, *P* value less than 0.05 was considered as statistically significant.

## Results

### CircNOL10 was down-regulated in BC tissues and cell lines

**Fig. 1:**
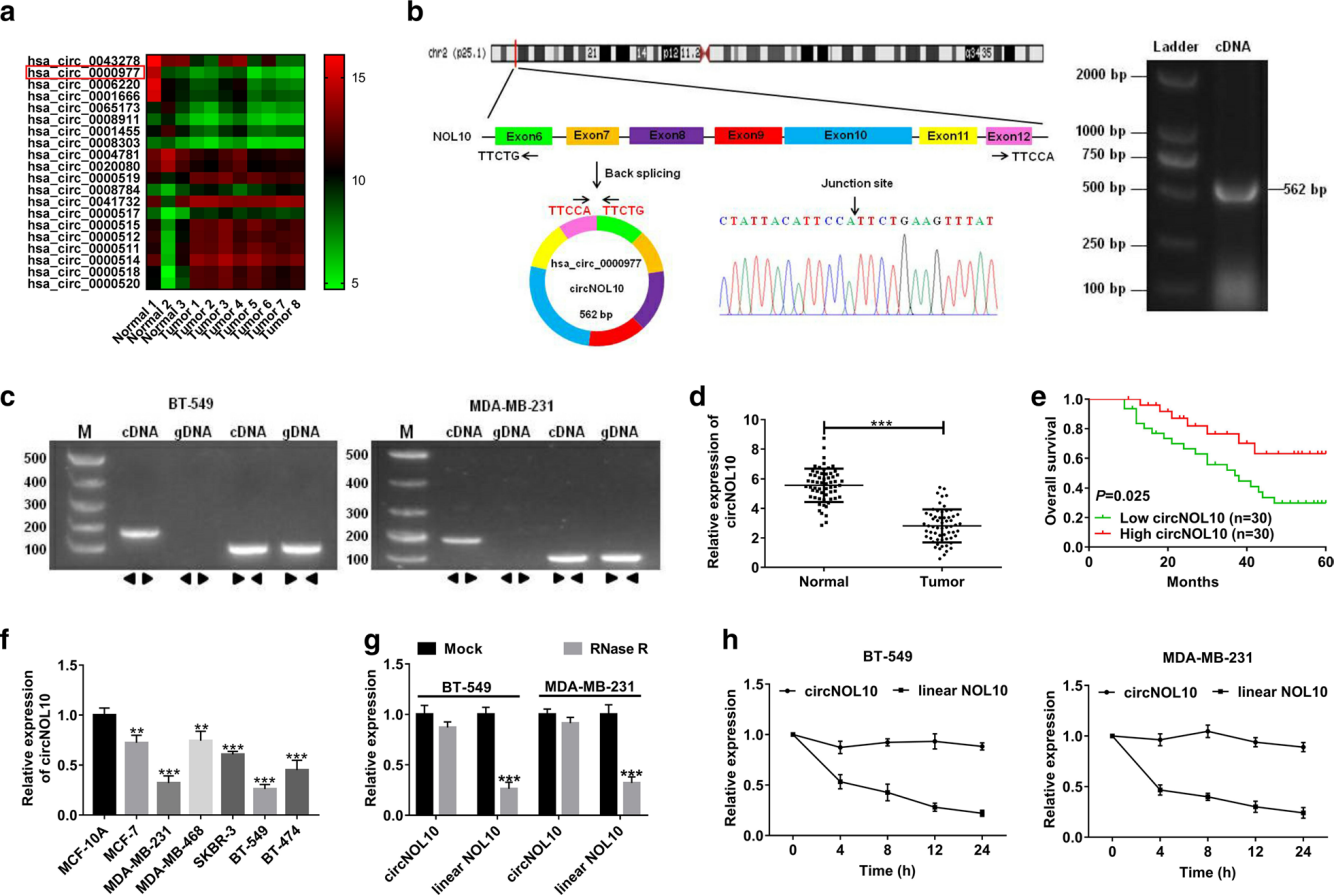
CircNOL10 is down-regulated in BC tissues and cell lines. **a** Heat map from GEO microarray database (GSE101123) revealed the top ten increased and decreased circRNAs in eight BC samples and three normal subjects. **b** The schematic diagram displaying that circNOL10 is back-spliced from NOL10 gene, ranging from the 6th exon to the 12th exon. The back-splice junction sites and RT-PCR product of circNOL10 were verified by Sanger sequencing and agarose gel electrophoresis, respectively. **c** RT-PCR was used to confirm the existence of circNOL10 and NOL10 from cDNA and gDNA in BC cells using the divergent and convergent primers, respectively. **d** The expression of circNOL10 in tumor tissues and adjacent normal tissues from 60 BC patients was tested by qRT-PCR. **e** The overall survival rate of patients with high or low circNOL10 expression was assessed via Kaplan–Meier survival analysis. **f** The expression of circNOL10 was measured in six BC cell lines and a normal breast epithelial cell line MCF-10A by qRT-PCR. **g** qRT-PCR analysis of circular and linear NOL10 expression in BT-549 and MDA-MB-231 cells with or without RNase R treatment. **h** qRT-PCR analysis was used to determine the effect of Actinomycin D on the levels of circNOL10 and linear NOL10 in BT-549 and MDA-MB-231 cells at indicated time points. **P < 0.01, ***P < 0.001

To identify abnormally expressed circRNAs in BC, GEO microarray database (GSE101123) containing eight BC samples and three normal samples were analyzed. The heatmap showed the top ten most down-regulated and up-regulated circRNAs (Fig. 1a). The 5 most down-regulated circRNAs (hsa_circ_0043278, hsa_circ_0000977, hsa_circ_0006220, hsa_circ_0001666 and hsa_circ_0065173) were selected and validated in 36 BC tumor tissues and adjacent non-cancerous tissues. As presented in Additional file 1: Figure S1, except for hsa_circ_0065173, the circRNAs showed a consistent expression level between the microarray database and qRT-PCR assay. Due to the function and mechanism of hsa_circ_0043278 and hsa_circ_0006220 (TADA2A) have been elucidated in a previous research [17], they were excluded for further study. From hsa_circ_000097 and hsa_circ_0065173, hsa_circ_000097 was selected for its more significant decrease in BC tumor tissues. Hsa_circ_0000977 is generated from NOL10 gene at exons 6 to 12 by back-splicing with a length of 562 bp (Fig. 1b), thus we term it circNOL10. To confirm the existence of circNOL10, the cDNA of circNOL10 was amplified and the head-to-tail splicing region was verified in the RT-PCR product of circNOL10 by Sanger sequencing (Fig. 1b). Also, the convergent primers and divergent primers were respectively designed to amplify the linear and circular transcripts of NOL10 by qRT-PCR, with cDNA and gDNA as the templates. As expected, circNOL10 was only amplified by divergent primers in cDNA but not in gDNA, while linear NOL10 was amplified by convergent primers in both cDNA and gDNA (Fig. 1c). Subsequently, we explored the expression of circNOL10 in sixty BC tissue samples and adjacent normal tissue subjects. As shown in Fig. 1d, circNOL10 was almost decreased by half in tumor tissues when compared to corresponding non-cancerous tissues. The relationship between circNOL10 and clinicopathological characteristics of BC patients was analyzed by the chi-squared test. Results showed that low expression of circNOL10 was associated with larger tumor size (p = 0.018), advanced TNM stage (p = 0.007), and lymph node metastasis (p = 0.038), but uncorrelated with age, menopausal, ER status, PR status, and HER-2 status (Table 1). Moreover, Kaplan–Meier survival analysis exhibited that patients with low circNOL10 expression displayed a shorter overall survival (Fig. 1e). Consistently, circNOL10 was confirmed to be reduced in BC cell lines in contrast to that in human normal mammary epithelial cell line MCF-10A (Fig. 1f). In addition, RNase R and Actinomycin D were used to examine the stability of circNOL10. The result found that RNase R treatment resulted in a significant degradation of linear NOL10, while circNOL10 expression was hardly changed (Fig. 1g). Also, circNOL10 displayed a longer half-life in response to Actinomycin D than linear NOL10 (Fig. 1h). These data indicated that circNOL10 may be associated with BC progression and prognosis.

**Table 1:** Correlation between circNOL10 expression and clinicopathological features in 60 breast cancer patients

Variable	circNOL10 expression		*P* value
	Low (n = 30)	High (n = 30)	
Age			0.573
≤ 50	10	8	
> 50	20	22	
Tumor size			0.018*
< 2 cm	8	17	
≥ 2 cm	22	13	
TNM stage			0.007*
I–II	14	24	
III–IV	16	6	
Lymph node metastasis			0.038*
Negative	10	18	
Positive	20	12	
Menopausal			0.438
Premenopausal	16	13	
Postmenopausal	14	17	
ER status			0.432
Negative	19	16	
Positive	11	14	
PR status			0.592
Negative	10	12	
Positive	20	18	
HER-2 status			0.584
Negative	9	11	
Positive	21	19	

The median expression level of circNOL10 was used as the cutoff

* *P* < 0.05 was considered as statistically significant

### CircNOL10 inhibited malignant cell behaviors in BC in vitro

**Fig. 2:**
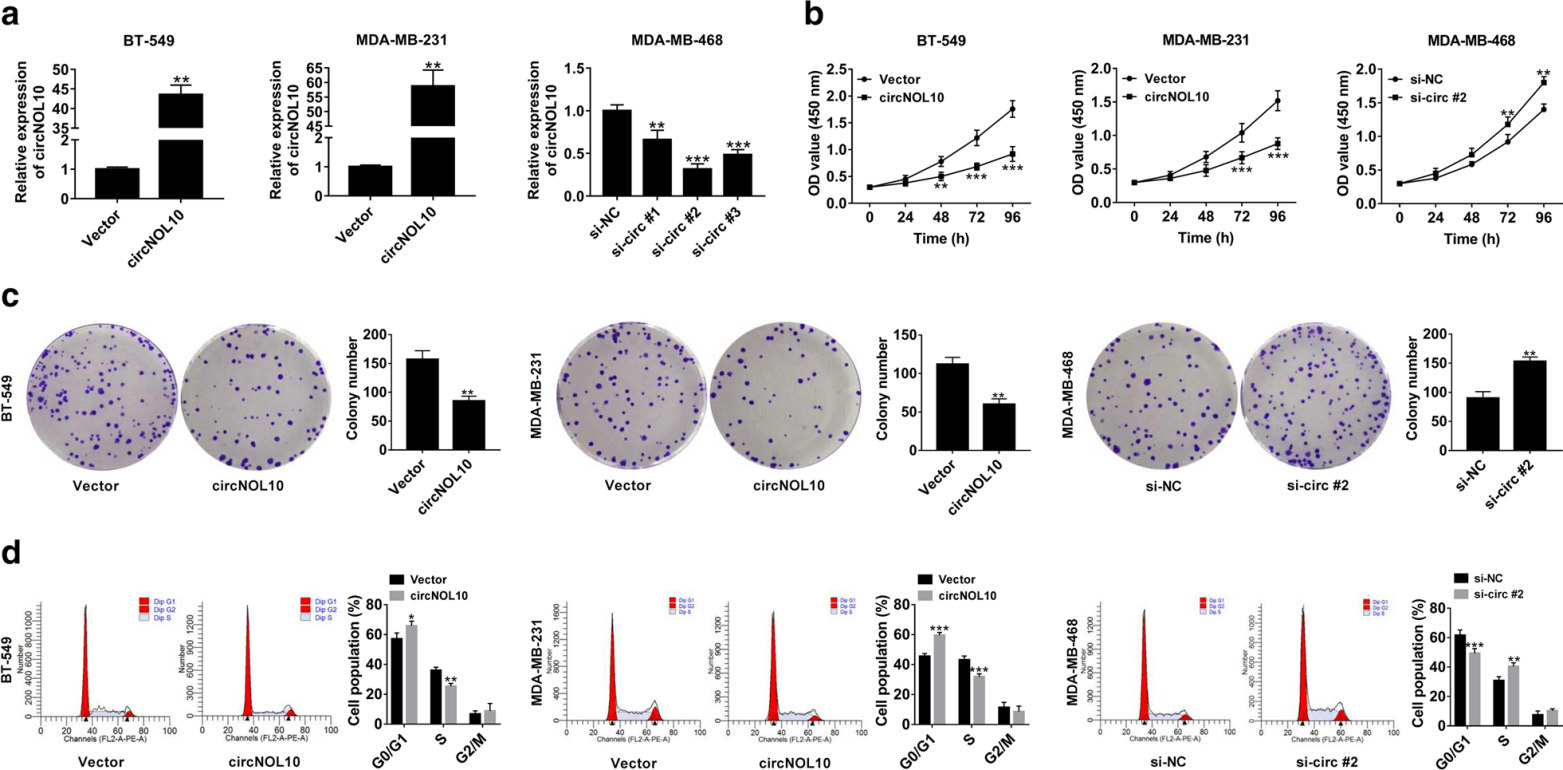
CircNOL10 suppresses BC cell proliferation. **a** qRT-PCR was conducted to determine the transfection efficiency of cicrNOL10-overexpressing vector (circNOL10) in BT-549 and MDA-MB-231 cells and siRNAs targeting circNOL10 (si-circ #1, si-circ #2 and si-circ #3) in MDA-MB-468 cells. **b**–**d** CCK-8 (**b**), plate clonality (**c**) and flow cytometry (**d**) assays were used to assess the influence of circNOL10 overexpression or knockdown on cell viability, colony forming ability and cell cycle distribution

**Fig. 3:**
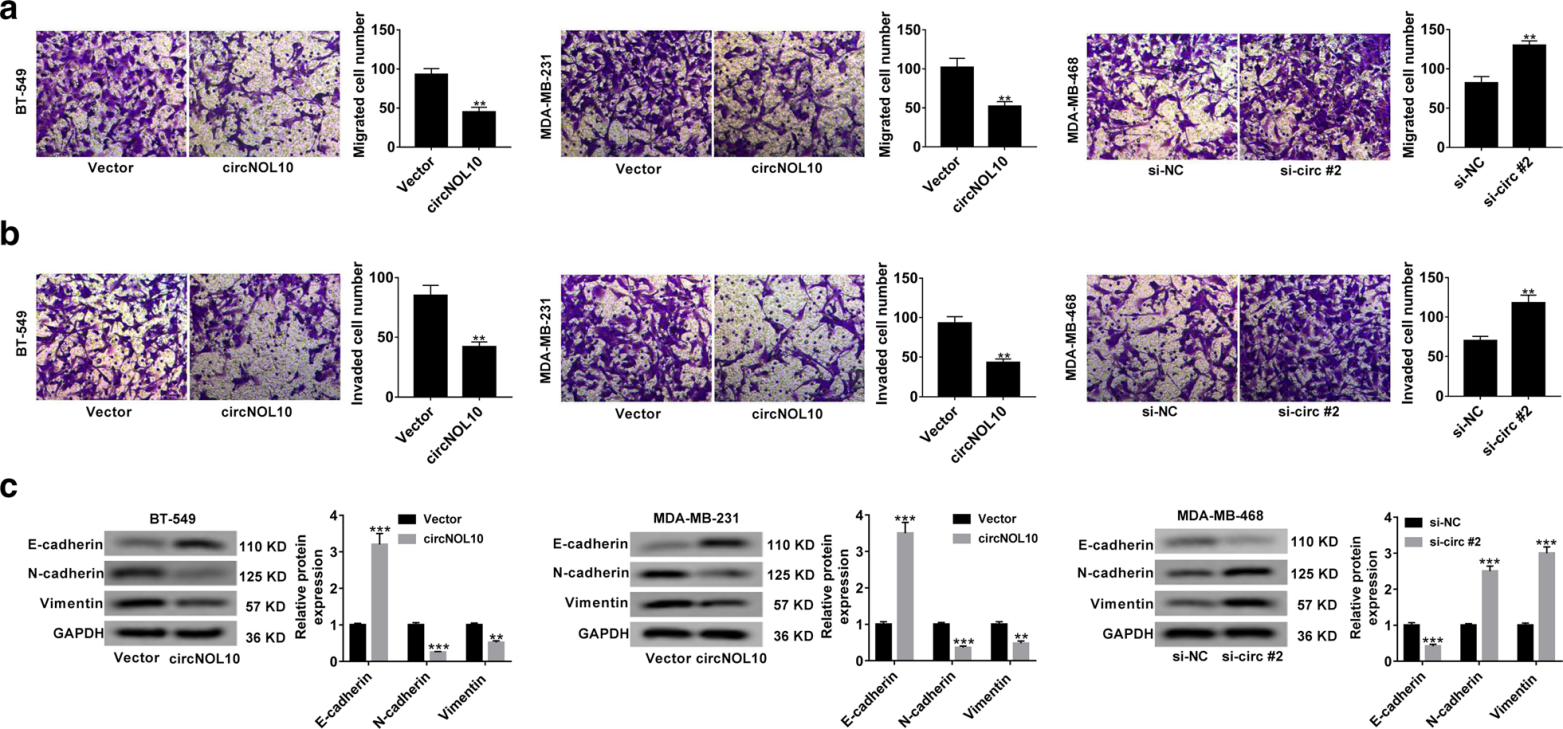
CircNOL10 inhibits cell migration, invasion and EMT in BC. **a**, **b** Transwell experiments were performed to detect cell migration and invasion capability in response to circNOL10 up-regulation or deficiency. **c** Western blot assays were performed to the measure the expression of EMT markers (E-cadherin, N-cadherin and vimentin) in BC cells transfected with circNOL10 or si-circ #2. *P < 0.05, **P < 0.01, ***P < 0.001

In order to explore the effects of circNOL10 in BC, circNOL10-overexpressing plasmid (circNOL10) was transfected into BT-549 and MDA-MB-231 cells, while si-RNAs targeting circNOL10 (si-circ #1, si-circ #2, and si-circ #3) were transfected into MDA-MB-468 cells. qRT-PCR found a significant increase of circNOL10 expression in BT-549 and MDA-MB-231 cells, and a striking down-regulation of circNOL10 expression in MDA-MB-468 cells (Fig. 2a). Si-circ #2 was selected for further experiments for its highest inference efficiency. CCK-8 assays revealed that overexpression of circNOL10 inhibited the viability of BT-549 and MDA-MB-231 cells, while silencing of circNOL10 enhanced the viability of MDA-MB-468 cells (Fig. 2b). Plate clonality assays demonstrated that the colony formation rate was apparently decreased in BT-549 and MDA-MB-231 cells transfected with circNOL10, but was increased in si-circNOL10-teansfeted MDA-MB-468 cells (Fig. 2c). Flow cytometry analysis proved that up-regulation of circNOL10 induced cell cycle arrest, and deficiency of circNOL10 promoted cell cycle progression (Fig. 2d). As presented by transwell experiments, enforced expression of circNOL10 resulted in a significant decline of cell migration and invasion, however, knockdown of circNOL10 led to an increase of cell metastasis (Fig. 3a, b). Western blot analysis was also performed to evaluate the influence of circNOL10 on epithelial-mesenchymal transition (EMT). The data manifested a rise of E-cadherin expression, and a reduction of N-cadherin and Vimentin expression in response to circNOL10 overexpression (Fig. 3c). Whereas, down-regulation of circNOL10 showed an opposite effect on the expression of epithelial and mesenchymal markers (Fig. 3c). In summary, circNOL10 suppressed BC cell proliferation, migration, invasion and EMT.

### CircNOL10 had no effects on its linear transcript

Some circRNAs, such as circGFRA1 and circ-TFF1, can bind to cognate linear transcripts to sequester mRNA from translation and thereby trigger the decrease of protein expression [18, 19]. Here, we found that linear NOL10 expression was significantly down-regulated in BC tumor tissues compared with that in adjacent normal tissues (Additional file 1: Figure S2A). However, NOL10 expression was not associated with the prognosis of BC patients (Additional file 1: Figure S2B). Moreover, no changed was observed in NOL10 mRNA and protein expression in response to circNOL10 overexpression or knockdown (Additional file 1: Figure S2C and D). Together, NOL10 was not the target gene of circNOL10.

### CircNOL10 served as a sponge for miR-767-5p in BC cells

**Fig. 4:**
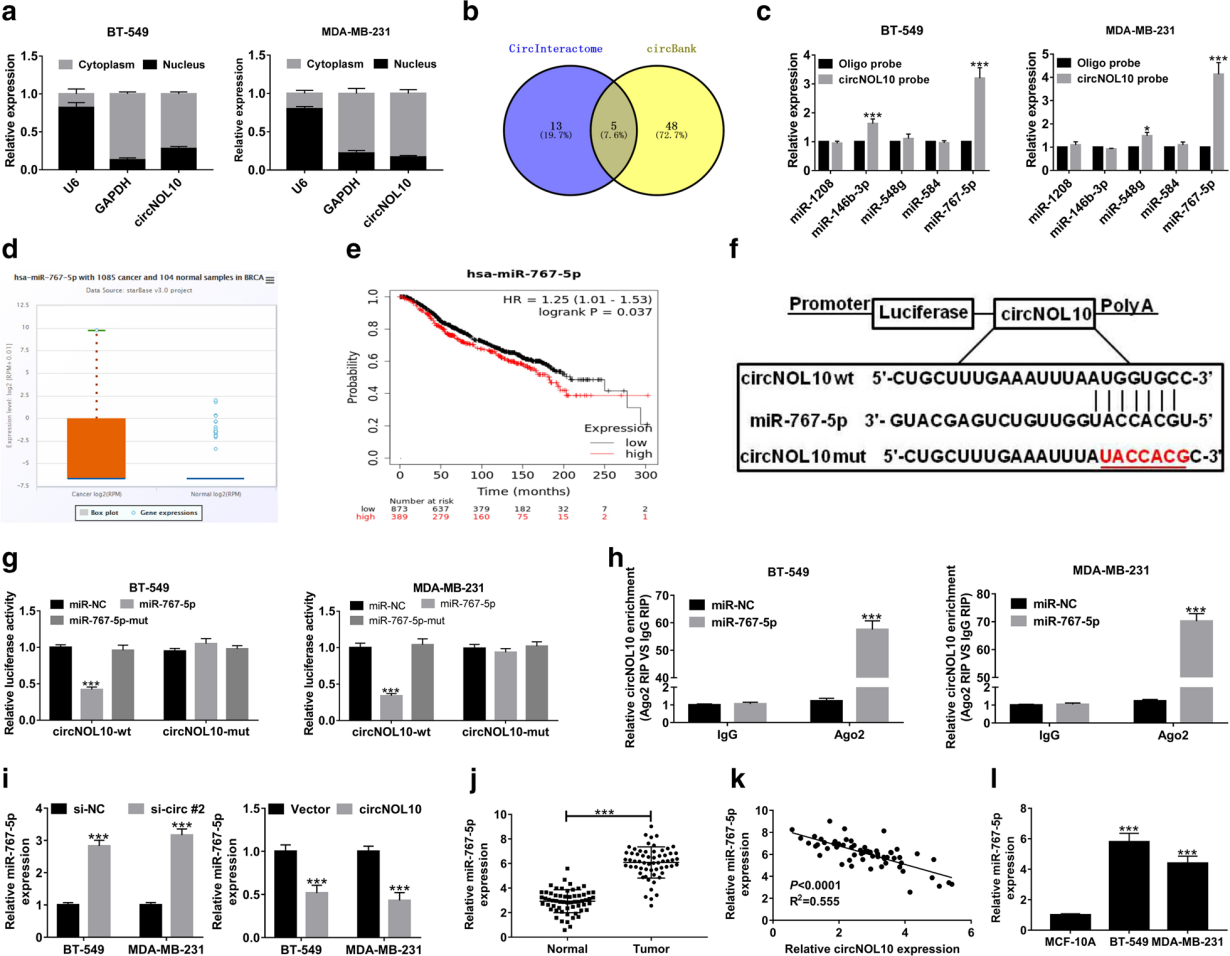
CircNOL10 acts as a sponge for miR-767-5p in BC cells. **a** Subcellular localization analysis was conducted to measure the percentage of circNOL10 expression in nucleus and cytoplasm of BC cells. **b** The candidate miRNAs containing the putative binding sites of circBOL10 were predicted via CircInteractome and CircBank online databases. **c** RNA pull down experiments were performed by using biotinylated circNOL10 probe, followed by qRT-PCR analysis of the candidate 5 miRNAs. **d** Expression level of miR-767-5p was analyzed in 1085 tumor tissues and 104 normal samples in breast invasive carcinoma (BRCA) by starBase Pan-Cancer Analysis Platform. **e** Kaplan–Meier survival analysis of TCGA cohort to examine the correlation between the overall survival and miR-767-5p expression in BC patients. **f** The predicted binding sequences between circNOL10 and miR-767-5p by web-based softwares. The red section is a sign of mutated bases. **g** The luciferase activities of circNOL10-wt and circNOL10-mut reporters in BT-549 and MDA-MB-231 cells transfected with miR-NC, miR-767-5p or miR-767-5p-mut were measured by dual-luciferase reporter assays. **h** RIP assays were conducted by using Ago2 or IgG antibody in BT-549 and MDA-MB-231 cells transfected with miR-NC or miR-767-5p, followed by the detection of circNOL10 enrichment in immunoprecipitates. **i** qRT-PCR was used to examine the level of miR-767-5p in BT-549 and MDA-MB-231 cells with circNOL10 knockdown or overexpression. **j** The level of miR-767-5p in 60 pairs of BC tissues and adjacent normal tissues. **k** Pearson’s correlation coefficients were used to assess the correlation between circNOL10 and miR-767-5p in BC tissues (n = 60). **l** Expression difference of miR-767-5p in BC and MCF-10A cells. ***P < 0.001

To gain an understanding of the molecular mechanisms of circNOL10 in regulating BC process, subcellular localization analysis was performed in BC cells. As exhibited in Fig. 4a, circNOL10 was mainly located in the cytoplasm of BT-549 and MDA-MB-213 cells. Given that one of the most popular mechanisms for circRNAs was serving as “miRNA sponges”, CircInteractome and circBank online databases were utilized to predict the potential target miRNAs of circNOL10. As displayed in Venn diagram, five candidate miRNAs (hsa-miR-1208, hsa-miR-146b-3p, hsa-miR-548g, hsa-miR-584, and hsa-miR-767-5p) were found to contain the putative binding sites on circNOL10 (Fig. 4b). Among them, miR-767-5p was the one that could be captured by circNOL10-specific probe in both BT-549 and MDA-MB-231 cells (Fig. 4c). According to the Pan-Cancer Analysis Platform of starBase v3.0, miR-767-5p expression was significantly higher in tumor tissues than that in normal samples in breast invasive carcinoma (BRCA) (Fig. 4d). Online KM plotter database (http://kmplot.com) displayed that high miR-767-5p expression was correlated with a poor clinical outcome of BC patients (Fig. 4e). Hence, miR-767-5p was chosen for further experiments. The wild-type circNOL10 sequences harboring the miR-767-5p targeting sites (circNOL10-wt) and its mutant version in which the miR-767-5p complementary sequences were mutated were inserted into the pmirGLO luciferase reporter (Fig. 4f). The dual-luciferase reporter assay manifested that overexpression of miR-767-5p contributed to a notable inhibition of luciferase activity of circNOL10-wt reporter in BT-549 and MDA-MB-231 cells compared with that in miR-NC group, however, the effect was disappeared when the binding sequences were mutated in miR-767-5p (Fig. 4g). Moreover, the luciferase activity of circNOL10-mut reporter was almost unchanged in all groups (Fig. 4g). RIP assays revealed that compared with miR-NC group, circNOL10 was significantly immunoprecipitated by Ago2 antibody in miR-767-5p overexpression group (Fig. 4h). Interestingly, miR-767-5p level was enhanced by the knockdown of circNOL10, while was suppressed upon circNOL10 overexpression (Fig. 4i). In addition, miR-767-5p expression was evidently increased in BC tissue samples and cell lines (Fig. 4j and l). The scatter diagram also presented that the level of miR-767-5p was negatively correlated with the level of circNOL10 in BC tumor tissues (Fig. 4k). Taken together, these results indicated that circNOL10 could serve as a sponge for miR-767-5p to decrease it expression in BC cells.

### CircNOL10 suppressed BC progression by sponging miR-767-5p in vitro

**Fig. 5:**
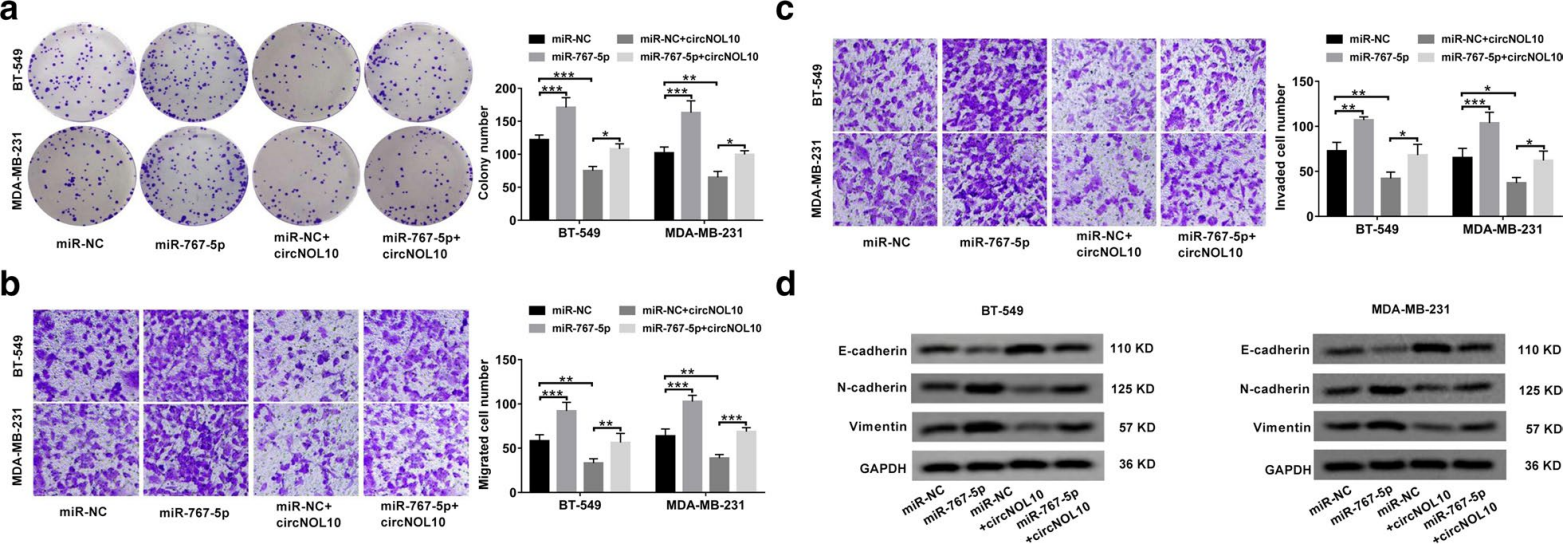
CircNOL10-mediated suppressive effects on BC cell malignant behaviors are reversed by miR-767-5p up-regulation. **a**–**d** BT-549 and MDA-MB-231 cells were transfected with miR-NC, miR-767-5p, miR-NC + circNOL10 or miR-767-5p + circNOL10. **a** Plate clonality assays were used to monitor the colony forming ability. **b**, **c** Transwell assays were performed to examine cell migration and invasion. **d** Western blot assays were applied to determine the protein expression of EMT-related genes including E-cadherin, N-cadherin and vimentin.*P < 0.05, **P < 0.01, ***P < 0.001

Next, we made an attempt to explore the functions of miR-767-5p in BC, and whether it was involved in the regulation of circNOL10 in BC. The results showed that overexpression of miR-767-5p led to an obvious promotion of cell proliferation (Fig. 5a), migration (Fig. 5b), invasion (Fig. 5c) and EMT (Fig. 5d), suggesting the carcinogenicity of miR-767-5p in BC. Moreover, circNOL10-mediated inhibition of cell proliferation, migration, invasion and EMT were largely counteracted by the restoration of miR-767-5p expression (Fig. 5a–d). Above all, circNOL10 exerted a tumor-suppressive effect in BC at least partly through acting as a sponge for miR-767-5p.

### CircNOL10 sponged miR-767-5p to induce SOCS2 expression and inactivate JAK/STAT signaling in BC cells

**Fig. 6:**
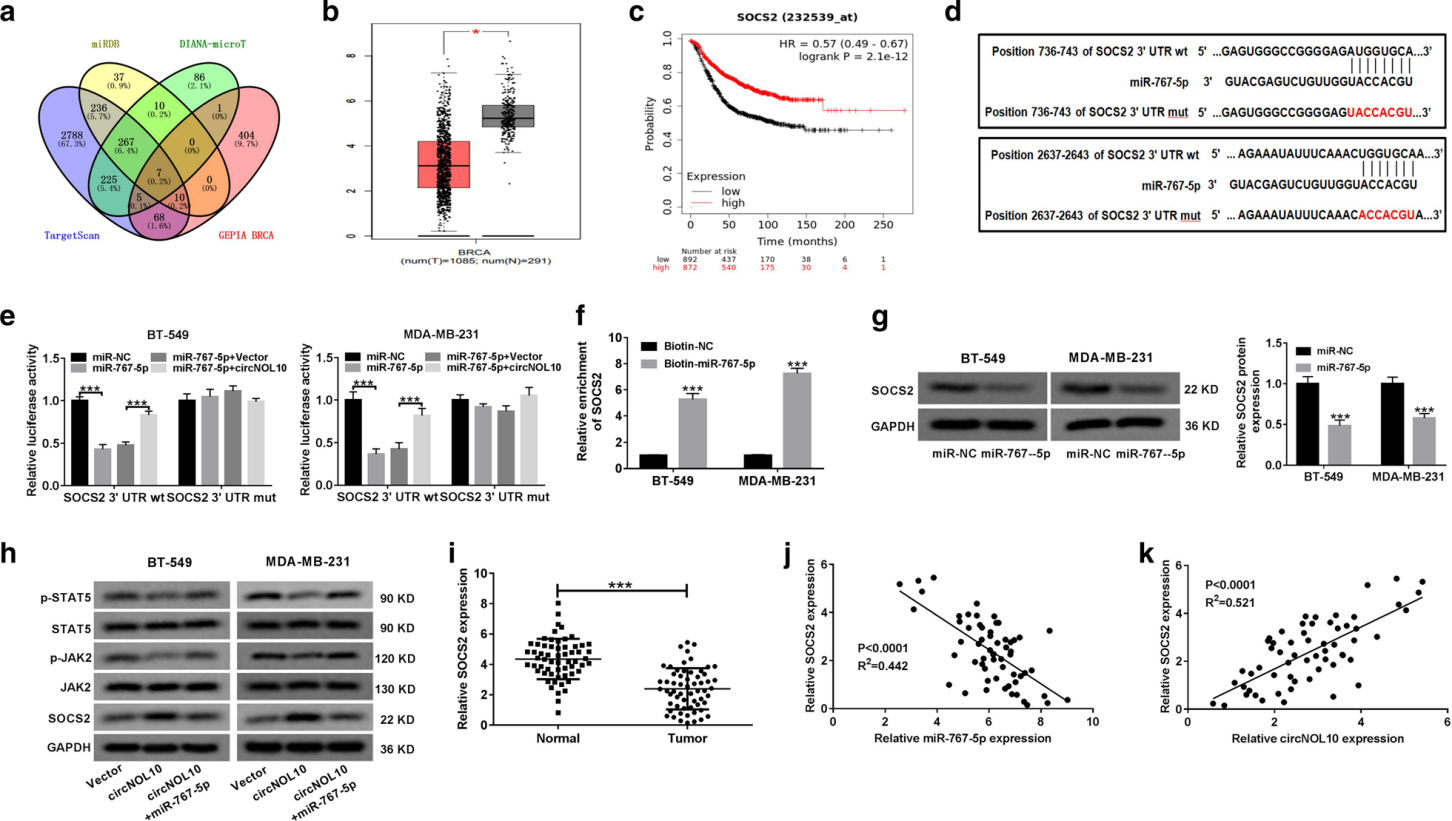
CircNOL10 regulates SOCS2 expression by sponging miR-767-5p in BC cells. **a** The potential candidate targets of miR-767-5p predicted by TargetScan, miRDB and DIANA-microT were further screened to search the down-regulated genes in BRCA by GEPIA database according to the criteria of log2 fold change < −2. **b** The expression difference of SOCS2 in 1085 BRCA tumor tissues and 291 normal tissues from GEPIA database. **c** The survival curves of BC patients with high or low expression of SOCS2 based on Kaplan–Meier plotter platform. **d** Diagram of the complementary binding sites between SOCS2 and miR-767-5p, as well as the mutant sequences of SOCS2 on miR-767-5p. **e** The relative luciferase activities of wild-type and mutant SOCS2 reporters in BT-549 and MDA-MB-231 cells transfected with miR-NC, miR-767-5p, miR-767-5p + Vector or miR-767-5p + circNOL10 were assessed via dual-luciferase reporter assay. **f** RNA pull-down assay was performed in BT-549 and MDA-MB-231 cells by using biotin-NC or biotin-miR-765-5p. **g** The effect of miR-767-5p overexpression on SOCS2 protein expression in BT-549 and MDA-MB-231 cells was examined by western blot assay. **h** The protein level of SOCS2, JAK2, p-JAK2, STAT5, and p-STAT5 was detected via western blot assay in BT-549 and MDA-MB-231 cells after transfection with vector, circNOL10 or circNOL10 + miR-767-5p. **i** qRT-PCR was used to monitor the level of SOCS2 mRNA in tumor tissues and adjacent non-cancerous samples from 60 BC patients. **j**, **k** The correlation between SOCS2 and miR-767-5p or circNOL10 was analyzed by Pearson test. ***P < 0.001

By virtue of three online databases TargetScan, miRDB, and DIANA-microT, we predicted 284 candidate targets of miR-767-5p. Among these target genes, only 7 (PI15, PGM5, CLDN11, SEC31B, SOCS2, HIF3A, HAS3) were found to be down-regulated in BRCA with log2 fold change lower than -2 according to GEPIA database (Fig. 6a). A previous report demonstrated that high SOCS2 expression could serve as an independent predictor for favorable prognosis in breast cancer [20]. Thus, SOCS2 was selected for further analysis. As displayed in Fig. 6b, SOCS2 expression was significantly lower in BRCA tumor tissues than that in normal samples. According to the TCGA data, an improved survival rate was observed in BC patient with high expression of SOCS2 (Fig. 6c). The two binding sites on SOCS2-3′UTR for miR-767-5p were exhibited in Fig. 6d. The dual-luciferase reporter experiments indicated that the luciferase activity of SOCS2-3′UTR wt reporter was remarkably declined in BT-549 and MDA-MB-231 cells transfected with miR-767-5p, but this effect was abrogated due to the overexpression of circNOL10 (Fig. 6e). However, no apparent fluctuation was observed in the luciferase activity of mutant reporter in any group (Fig. 6e). Also, as manifested by biotin-labelled RNA pull-down assay, SOCS2 mRNA was significantly enriched by Biotin-miR-767-5p rather than Biotin-NC (Fig. 6f). In BT-549 and MDA-MB-231 cells, the protein level of SOCS2 was strikingly reduced by miR-767-5p overexpression (Fig. 6g). These data confirmed SOCS2 as a downstream target of miR-767-5p.

It is acknowledged that one of the potential action modes for circRNAs is serving as sponges for miRNAs to prevent their degradation on target mRNAs [21]. Herein, we further investigated whether circNOL10 could regulate SOCS2 expression via sponging miR-767-5p. As shown in Fig. 6h, the protein level of SOCS2 was distinctly increased in BT-549 and MDA-MB-231 cells transfected with circNOL10, while this promotive effect was reversed due to re-introduction of miR-767-5p. SOCS2 has been considered as important regulators of the Janus kinase/signal transducers and activators of transcription (JAK-STAT) pathway in various inflammatory and neoplastic diseases [22]. Here, we found that overexpression of circNOL10 resulted in an evident decrease of p-JAK2 and p-STAT5 level, but this inhibitory effect was abated by the restoration of miR-767-5p expression to a great extent (Fig. 6h). Consistent with the predictive data, qRT-PCR results verified the down-regulation of SOCS2 expression in BC tissue specimens (Fig. 6i). What’s more, the level of SOCS2 was negatively associated with miR-767-5p expression, and positively correlated with circNOL10 expression in BC tumor tissues (Fig. 6j and k). Collectively, circNOL10 acted as a sponge of miR-767-5p to up-regulate SOCS2 expression and inactivate JAK2/STAT5 signaling in BC cells.

### Overexpression of SOCS2 inhibited BC progression in vitro

**Fig. 7:**
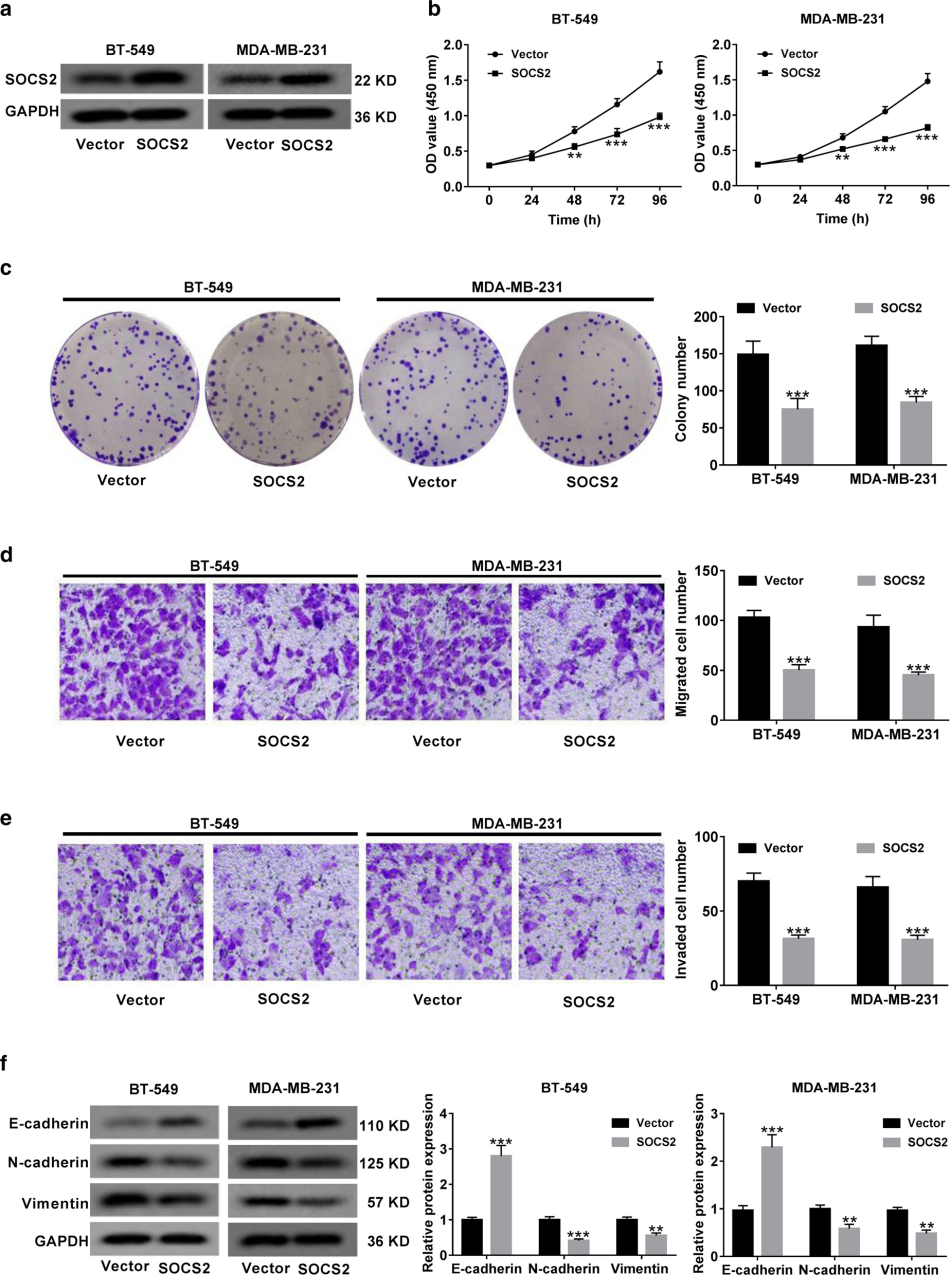
SOCS2 overexpression inhibits BC cell proliferation, migration, invasion and EMT. **a** SOCS2-overexpressing plasmid (SOCS2) was transfected into BT-549 and MDA-MB-231 cells, followed by western blot analysis of SOCS2 protein level. **b** CCK-8 assays were used to determine the effect of SOCS2 on cell viability. **c** Colony forming assays were performed in BT-549 and MDA-MB-231 cells with SOCS2 overexpression. **d**, **e** Transwell assays were applied to evaluate the migration and invasion abilities of SOCS2-overexpressing cells. **f** Western blot analysis of E-cadherin, N-cadherin and vimentin expressions in SOCS2-transfected cells. **P < 0.01, ***P < 0.001

Next, we further explored the biological function of SOCS2 in BC progression by transfecting SOCS2-overexpression plasmid (SOCS2) into BT-549 and MDA-MB-231 cells. The transfection efficiency was confirmed by western blot (Fig. 7a). Functional experiments elucidated that overexpression of SOCS2 led to a dramatic inhibition of cell viability (Fig. 7b), colony formation (Fig. 7c), migration (Fig. 7d), invasion (Fig. 7e) and EMT (Fig. 7f). To be concluded, SOCS2 exerted a tumor-suppressive role in BC.

### Knockdown of SOCS2 reversed circNOL10-mediated antineoplastic property in vitro

**Fig. 8:**
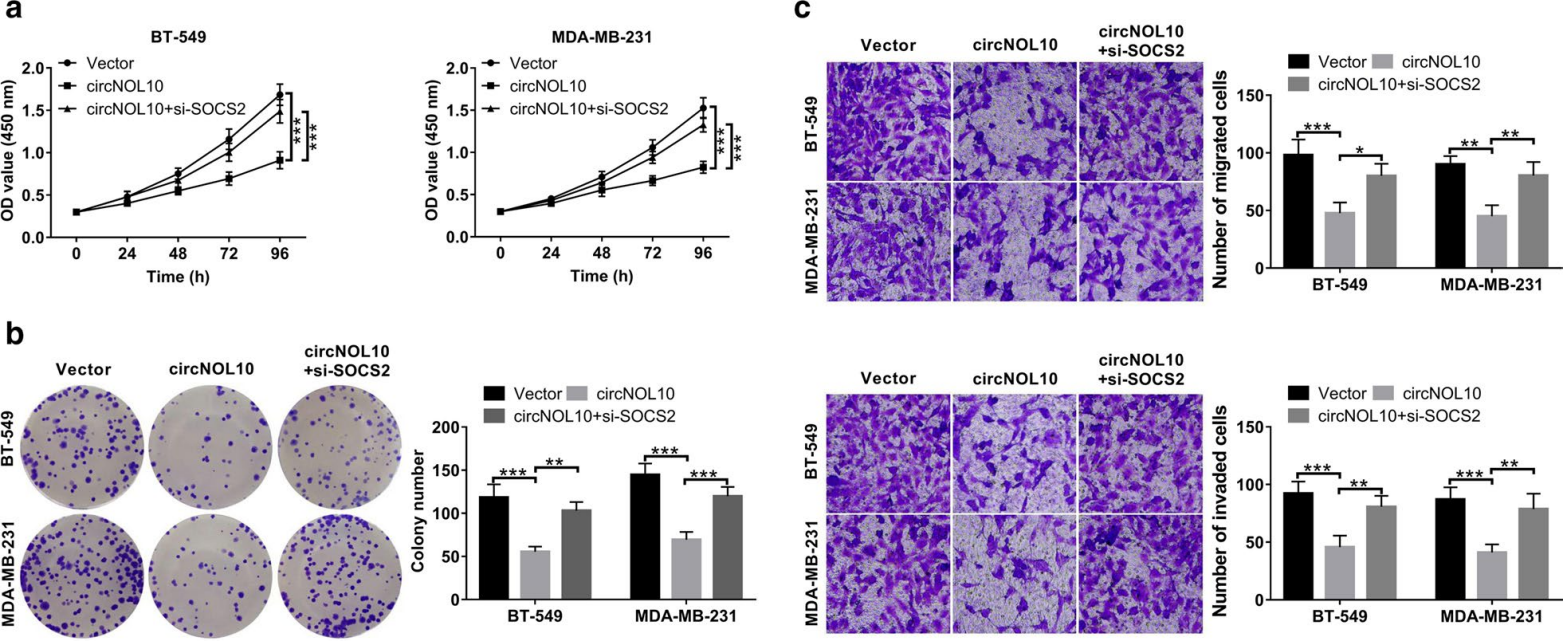
circNOL10 suppresses BC cell proliferation and metastasis via up-regulating SOCS2 expression. BT-549 and MDA-MB-231 cells were transfected with vector, circNOL10 or circNOL10 + si-SOCS2. **a** CCK-8 assays of cell viability at indicated time points. **b** Colony forming assays for transfected BC cells. **c**, **d** Transwell assays were utilized to examine cell migration and invasion

To further address whether circNOL10 suppressed BC cell malignant phenotypes via regulating SOCS2 expression, BT-549 and MDA-MB-231 cells were co-transfected with circNOL10-overexpression vector (circNOL10) and si-SOCS2. The results manifested that circNOL10-triggered anti-proliferative role was attenuated due to the down-regulation of SOCS2 (Fig. 8a, b). Likewise, circNOL10-mediated inhibition of cell migration and invasion was greatly eliminated with the decrease of SOCS2 expression (Fig. 8c, d). These data revealed that circNOL10 repressed BC cell proliferation and metastasis via promoting SOCS2 expression.

### CircNOL10 repressed xenograft tumor growth in vivo

**Fig. 9:**
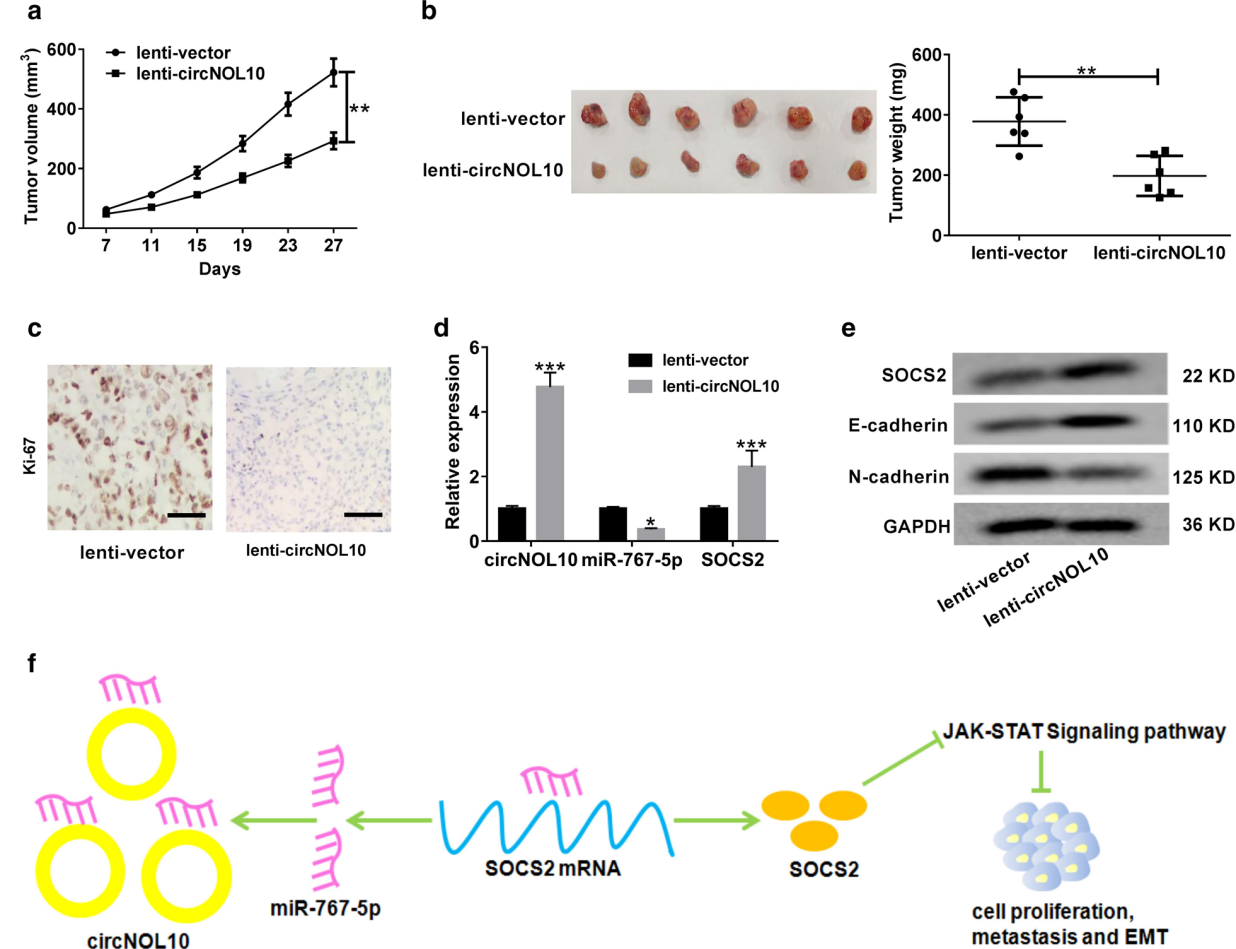
CircNOL10 suppresses tumor growth in vivo. BT-549 cells infected with lenti-vector or lenti-circNOL10 were subcutaneously injected into nude mice. **a** The tumor volumes were monitored every 4 days. **b** At 27 days post-inoculation, mice were killed and tumor weights were measured. **c** IHC staining was applied to evaluate the protein expression of proliferation index Ki-67 in the excised tumors. Scale bar, 50 μm. **d** qRT-PCR analysis of circNOL10, miR-767-5p and SOCS2 in xenograft tumors. **e** Western blot analysis of SOCS2, E-cadherin and N-cadherin proteins in tumor masses. **f** The schematic diagram displays that circNOL10 sponges miR-767-5p to protect SOCS2 from miR-767-5p-induced degradation, thus inactivating JAK/STAT signaling and suppressing BC progression. *P < 0.05, **P < 0.01, **P < 0.01

To observe the effect of circNOL10 on tumor growth in vivo, BT-549 cells infected with lenti-vector or lenti-circNOL10 were injected into nude mice. The results showed that the volume and weight of xenograft tumors were dramatically reduced in circNOL10 group compared to that in vector group (Fig. 9a, b). Accordantly, IHC analysis showed that overexpression of circNOL10 lowered the level of proliferation marker Ki-67 in excised tumors compared with the vector group (Fig. 9c). Moreover, the expressions of circNOL10 and SOCS2 were increased, while miR-767-5p expression was decreased in tumors derived from circNOL10-overexpressing cells (Fig. 9d). As demonstrated by western blot, up-regulation of circNOL10 led to a rise of SOCS2 and E-cadherin protein levels, while a decline of N-cadherin protein expression in tumor tissues (Fig. 9e). To sum up, circNOL10 slowed BC tumor growth in vivo.

## Discussion

In recent years, it is acknowledged that circRNAs play vital regulatory roles in the genesis and development of human tumors, suggesting their potential as prognostic biomarkers and therapeutic targets in cancer [23]. Through analyzing the GEO microarray database (GSE101123) and performing qRT-PCR, we focused on circNOL10 to explore its biological functions and action mechanisms in BC tumorigenesis. Our findings reveal a novel circNOL10-miR-767-5p-SOCS2-JAK/STAT regulatory pathway in BC progression.

In the current research, circNOL10 expression was validated to be decreased in BC tumor tissues and cells. Decreased circNOL10 expression was correlated to larger tumor size, advanced TNM stage, lymph node metastasis and unfavorable prognosis. Functionally, circNOL10 overexpression repressed BC cell proliferation, migration, invasion and EMT, while circNOL10 knockdown displayed contrary tendency. Moreover, overexpression of circNOL10 slowed xenograft tumor growth in vivo. These results disclosed the antineoplastic role of circNOL10 in BC progression. Congruent with our data, Nan et al. reported that circNOL10 was down-regulated in lung cancer, and circNOL10 exerted anti-cancer effects via transcriptional regulation of the HN polypeptide family by SCML1 [12]. Zhang et al. recently disclosed that circNOL10 repressed cell proliferation and metastasis in colorectal cancer by regulating KLF9 expression via sponging miR-135a/b-5p [24].

Emerging evidence proposed the ceRNA mechanism for circRNAs, that is to say, circRNAs could serve as sponges of miRNAs to prevent miRNAs-induced inhibition on the expression and activity of target genes [25]. For instance, circ-UBE2D2 contributed to BC cell proliferation, migration, and invasion through functioning as the sponge of miR-1236 and miR-1287 [26]. CircHIPK3 overexpression resulted in an evident reduction of aggressiveness and metastasis in bladder cancer via regulating miR-558/heparanase axis [27]. Thus, we further investigated whether circNOL10 exerted anti-tumor role in BC by the similar circRNA-miRNA-mRNA pathway. Through subcellular fractionation, we found that circNOL10 was mostly located in the cytoplasm. According to the data from online programs, RNA pull-down, luciferase reporter, and RIP assays, miR-767-5p was confirmed as a downstream target of circNOL10 and was degraded by circNOL10. As reported by Li et al. [28], if the circRNA-protein complex digests its target miRNAs, overexpression of this circRNA would reduce the expression of these miRNAs. However, if the circRNA only binds to the miRNA and inhibits its function, the expression of the miRNA would not be affected. Here, we found that the action mode of circNOL10 was consistent with the former theoretical ideas. However, under what circumstances will a circRNA digest or reserve the target miRNAs remain needs in-depth exploration.

MiR-767-5p was previously elucidated as an oncogenic factor in several human malignancies, such as melanoma [29], multiple myeloma [30, 31], prostate cancer [32], hepatocellular carcinoma (HCC) [33] and thyroid cancer [34]. Nevertheless, there is still a lack of investigation on the biological significance of miR-767-5p in BC. In this study, miR-767-5p was proved to be highly expressed in BC tissues and cells. And, miR-767-5p expression was inversely correlated with circNOL10 expression in BC tumor tissues. Moreover, enforced expression of miR-767-5p promoted cell proliferation, migration, invasion, and EMT, highlighting the carcinogenesis of miR-767-5p in BC. Furthermore, circNOL10-mediated suppressive effect on BC was greatly reversed by the ectopic expression of miR-767-5p. To sum up, circNOL10 inhibited BC progression partly through sponging miR-767-5p.

In the follow-up exploration, suppressor of cytokine signaling 2 (SOCS2) was verified as a direct target of miR-767-5p. Suppressor of cytokine signaling (SOCS) protein family, such as SOCS1 and SOCS3, is reported to be closely correlated with cancer cell proliferation and cancer-associated inflammation [35]. SOCS2, another member of the SOCS family, displays both oncogenic and tumor-suppressive activities in different human tumors [22]. For example, a document exhibited that SOCS2 expression was decreased in hepatocellular carcinoma, and overexpression of SOCS2 repressed the metastatic potential of hepatocellular carcinoma cells in vivo and in vitro [36]. On the contrary, SOCS2 was elucidated as a growth promoter in acute myeloid leukemia [37]. Moreover, SOCS2 seems to have a dual effect in prostate cancer. Hoefer et al. uncovered the growth-promoting role for SOCS2 and offered an explanation for a high SOCS2 expression in malignant tissues upon androgenic stimulation [38]. However, Das et al. unveiled that low levels of SOCS2 was strongly linked to disease recurrence and metastasis in clinical specimens, and SOCS2 overexpression inhibited metastatic features of prostate cancer cells [39]. Conflicting data for SOCS2 were also observed in colorectal cancer (CRC) [40, 41]. Differences between SOCS2 mRNA and protein levels might be attributed to the active degradation of SOCS2 protein [42]. In BC, SOCS2 were found to be significantly down-regulated in tumor tissues compared with the corresponding adjacent non-cancerous tissues [43]. Loss of SOCS2 might be associated with cell proliferation, tumor growth, and a poor prognosis in breast carcinoma [20, 44]. However, the detailed function relevance of SOCS2 with BC is still obscure. Here, we validated that SOCS2 expression was reduced in BC, and overexpression of SOCS2 suppressed BC cell proliferation, migration, invasion and EMT. These data provide evidence for SOCS2 as a tumor-suppressor in BC. More importantly, circNOL10 could positively modulate SOCS2 expression in BC cells via acting as a sponge of miR-767-5p. Knockdown of SOCS2 reversed circNOL10-induced antineoplastic property in BC cells. Together, circNOL10 exerted tumor-suppressive role in BC through regulating SOCS2.

JAK/STAT signaling has become a favorite target for drug development and cancer therapy for its important involvement in tumor cell survival, proliferation and invasion [45]. SOCS proteins are regarded as inducible inhibitors of cytokine receptors that could activate JAK-STAT pathway [46]. Herein, we found that circNOL10 inactivated JAK2/STAT5 signaling by sponging miR-767-5p and up-regulating SOCS2. All these results draw us to conclude that circNOL10 inhibited BC progression partly through regulating miR-767-5p/SOCS2/JAK/STAT axis (Fig. 9f).

## Conclusion

In conclusion, circNOL10 sponges miR-767-5p to facilitate SOCS2 expression and inactivate JAK2/STAT5 signaling, thus inhibiting BC cell proliferation and metastasis. This result may provide a new insight into the molecular mechanism of circNOL10 in BC and highlight that targeting circNOL10/miR-767-5p/SOCS2 may be a prospective therapeutic option for BC. However, more animal experiments and clinical investigations are warranted for the immediate human application of circNOL10.

## Supplementary Information

**Additional file 1: Table S1.** Primer sequences used in qRT-PCR. **Figure S1.** qRT-PCR assays of hsa_circ_0043278, hsa_circ_0000977, hsa_circ_0006220, hsa_circ_0001666 and hsa_circ_0065173 in 36 paired BC tumor tissues and adjacent normal tissues. **Figure S2.** CircNOL10 had no effects on its linear transcript. (A) qRT-PCR analysis of NOL10 expression in tumor tissues and adjacent non-neoplastic tissues from 60 patients. (B) The correlation between NOL10 expression and overall survival rate in BC patients. (C and D) qRT-PCR and western blot assays were performed to examine the effect of circNOL10 overexpression or knockdown on NOL10 mRNA and protein expression in BC cells. ***P < 0.001; N.S., not significant.

**Additional file 2: Figure S3.** C-BT-549 cell. C-MDA-MB-231-cell. C-MDA-MB-268-cell. **Figure S5.** D-BT-549 cell. D-MDA-MB-231 cell. **Figure 6.** G-BT-549 cell. **Figure 7.** A-BT-549 cell

## Data Availability

The data sets used and/or analyzed during the current study are available from the corresponding author on reasonable request.
