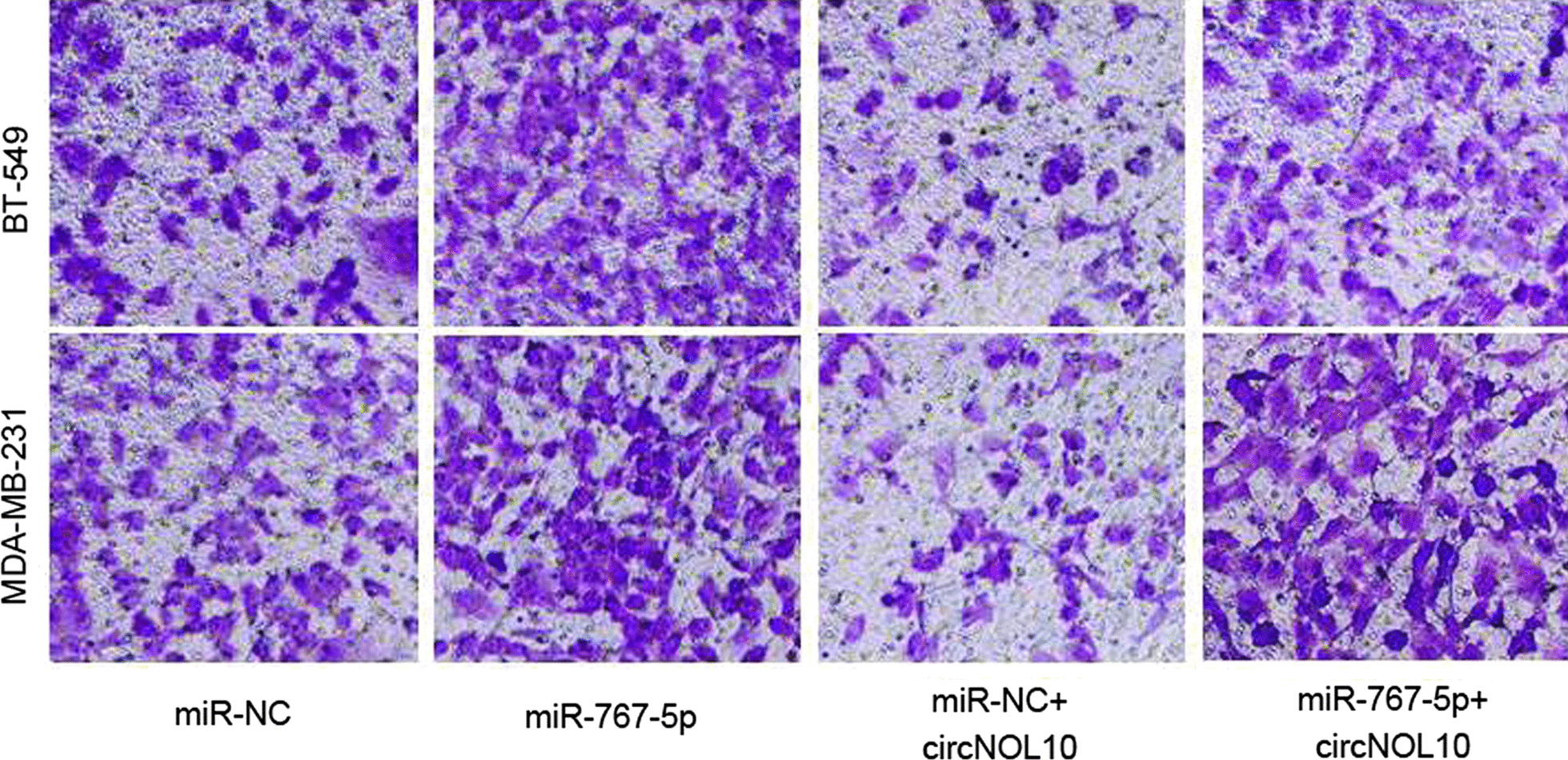# Correction to: CircNOL10 suppresses breast cancer progression by sponging miR-767-5p to regulate SOCS2/JAK/STAT signaling

**DOI:** 10.1186/s12929-021-00723-9

**Published:** 2021-04-26

**Authors:** Fang Wang, Xiaochun Wang, Jingruo Li, Pengwei Lv, Mingli Han, Lin Li, Zhuo Chen, Lingling Dong, Nan Wang, Yuanting Gu

**Affiliations:** 1 Department of Breast Surgery, The First Affiliated Hospital of Zhengzhou University, No.1 Jianshe East Road, Erqi, Zhengzhou, 450000 China; 2 Department of Breast Surgery, Affiliated Hospital of Hebei University, Baoding, 071000 China

## Correction to: J Biomed Sci (2021) 28:4 https://doi.org/10.1186/s12929-020-00697-0

Following publication of the original article [1], Fig. 5b was found to be incorrect. The image for MDA-MB-231 cells in miR-NC group was unintentionally used for the miR-767-5p + circNOL10 group. The corrected Fig. 5b is given. The original paper has been updated.