# Supplementary material for: CircNOL10 suppresses breast cancer progression by sponging miR-767-5p to regulate SOCS2/JAK/STAT signaling
Source: J Biomed Sci. 2021 Jan 4;28:4. doi: 10.1186/s12929-020-00697-0 (PMC7780627; doi:10.1186/s12929-020-00697-0)
Supplement: Supplementary file 1 — Additional file 1: Table S1. Primer sequences used in qRT-PCR. Figure S1. qRT-PCR assays of hsa_circ_0043278, hsa_circ_0000977, hsa_circ_0006220, hsa_circ_0001666 and hsa_circ_0065173 in 36 paired BC tumor tissues and adjacent normal tissues. Figure S2. CircNOL10 had no effects on its linear transcript. (A) qRT-PCR analysis of NOL10 expression in tumor tissues and adjacent non-neoplastic tissues from 60 patients. (B) The correlation between NOL10 expression and overall survival rate in BC patients. (C and D) qRT-PCR and western blot assays were performed to examine the effect of circNOL10 overexpression or knockdown on NOL10 mRNA and protein expression in BC cells. ***P < 0.001; N.S., not significant. [file 12929_2020_697_MOESM1_ESM.docx]

**Table S1 Primer sequences used in qRT-PCR**

| Gene | Forward Primer (5’-3’) |
| --- | --- |
| circNOL10 | F: CAACTCAGGCATGCTTCTGA |
|  | R: TATGGTTCCTGTGGCAAACA |
| circNOL10 (PCR) | F: CTATTACATTCCATTCTGAA |
|  | R: ATGCCCATCTTGGGGGTTTC |
| NOL10 | F: GGAGAATAATGTTTGTGACA |
|  | R: TCTGTTTCGAGTTCTTGGGT |
| miR-767-5p | F: CTCAACTGGTGTCGTGGAGTCGGCAATTCAGTTGAGCATGCTCAG |
|  | R: ACACTCCAGCTGGGTGCACCATGGTTGTCTGAG |
| SOCS2 | F: TTAAAAGAGGCACCAGAAGGAAC |
|  | R: AGTCGATCAGATGAACCACACT |
| GAPDH | F: TGTTCGTCATGGGTGTGAAC |
|  | R: ATGGCATGGACTGTGGTCAT |
| U6 | F: ATTGGAACGATACAGAGAAGATT |
|  | R: GGAACGCTTCACGAATTTG |

**Figure S1**

**
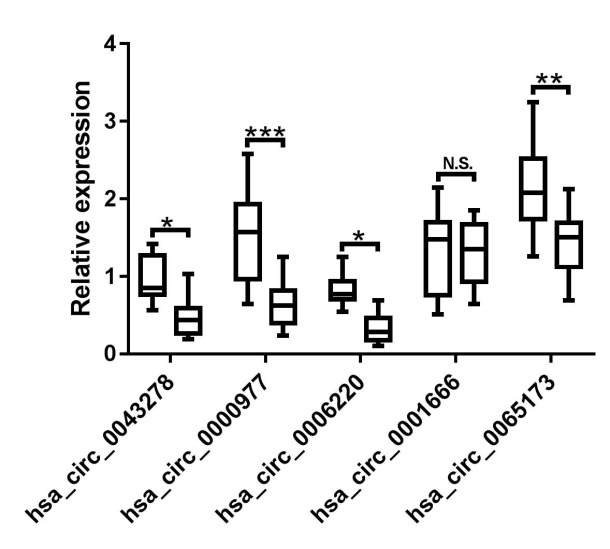
**

**Figure S1 qRT-PCR assays of hsa_circ_0043278, hsa_circ_0000977, hsa_circ_0006220, hsa_circ_0001666 and hsa_circ_0065173 in 36 paired BC tumor tissues and adjacent normal tissues.**

**Figure S2**


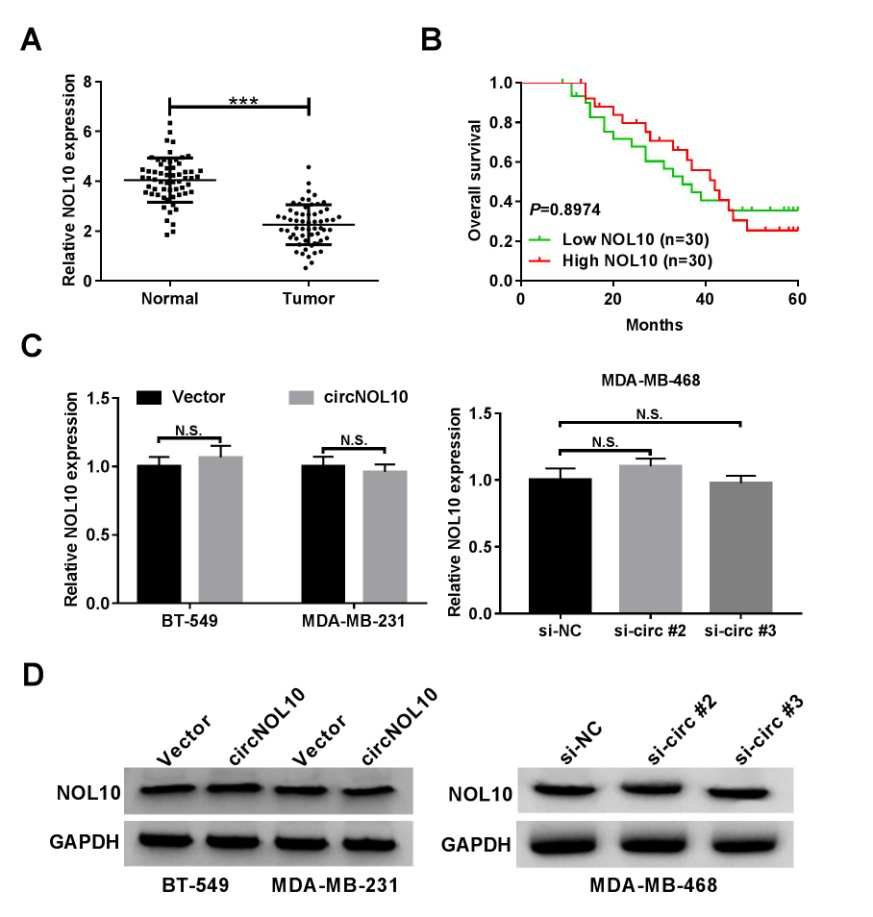


**Figure S2 CircNOL10 had no effects on its linear transcript.** (A) qRT-PCR analysis of NOL10 expression in tumor tissues and adjacent non-neoplastic tissues from 60 patients. (B) The correlation between NOL10 expression and overall survival rate in BC patients. (C and D) qRT-PCR and western blot assays were performed to examine the effect of circNOL10 overexpression or knockdown on NOL10 mRNA and protein expression in BC cells. ****P* < 0.001; N.S., not significant.
