# Supplementary material for: CircNOL10 suppresses breast cancer progression by sponging miR-767-5p to regulate SOCS2/JAK/STAT signaling
Source: J Biomed Sci. 2021 Jan 4;28:4. doi: 10.1186/s12929-020-00697-0 (PMC7780627; doi:10.1186/s12929-020-00697-0)
Supplement: Supplementary file 2 — Additional file 2: Figure S3. C-BT-549 cell. C-MDA-MB-231-cell. C-MDA-MB-268-cell. Figure S5. D-BT-549 cell. D-MDA-MB-231 cell. Figure 6. G-BT-549 cell. Figure 7. A-BT-549 cell [file 12929_2020_697_MOESM2_ESM.docx]

**Figure 3C-BT-549 cell**

**GAPDH**


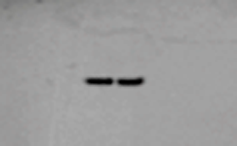


**E-cadherin**

**
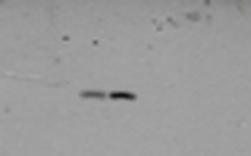
**

**N-cadherin**


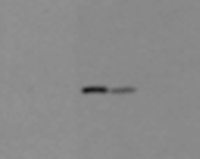


**Vimentin**

**
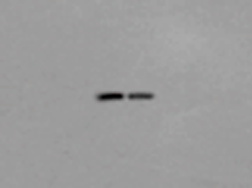
**

**Figure 3C-MDA-MB-231-cell**

**GAPDH**

**
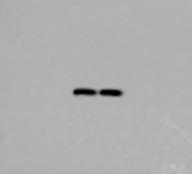
**

**E-cadherin**

**
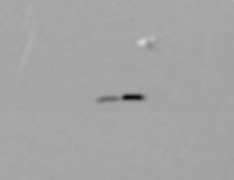
**

**N-cadherin**

**
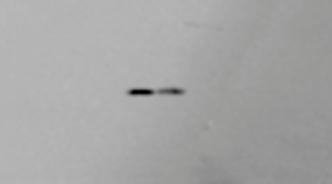
**

**Vimentin**

**
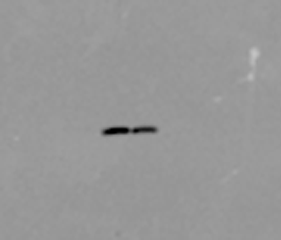
**

**Figure 3C-MDA-MB-268-cell**

**GAPDH**

**
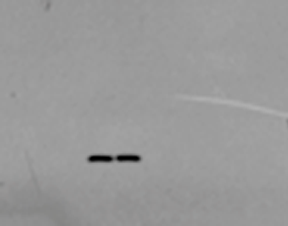
**

**E-cadherin**

**
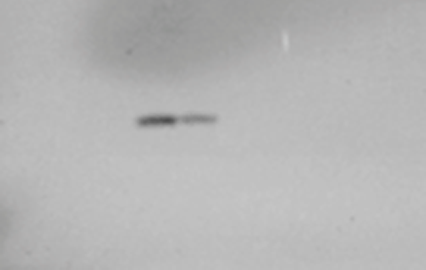
**

**N-cadherin**

**
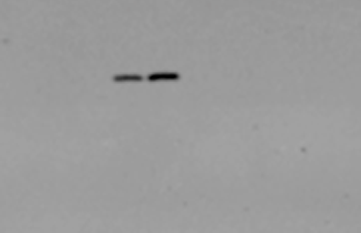
**

**Vimentin**

**
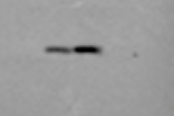
**

**Figure 5D-BT-549 cell**

**GAPDH**

**
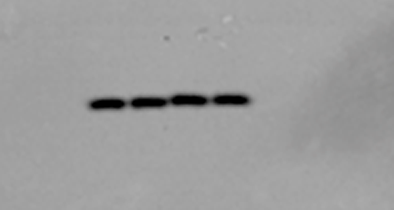
**

**E-cadherin**

**
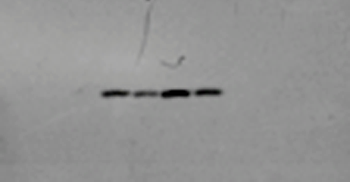
**

**N-cadherin**

**
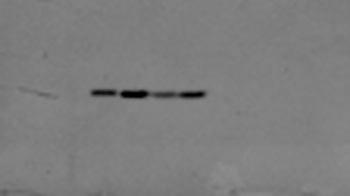
**

**Vimentin**

**
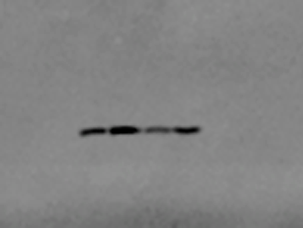
**

**Figure 5D-MDA-MB-231 cell**

**GAPDH**

**
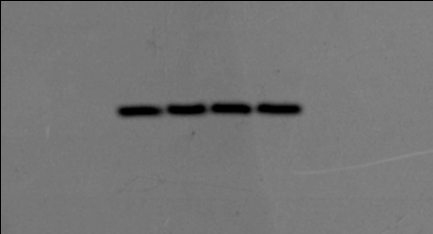
**

**E-cadherin**

**
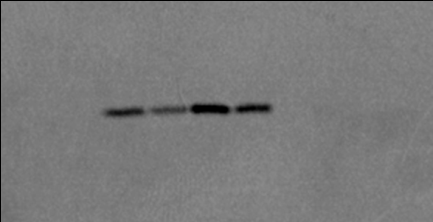
**

**N-cadherin**

**
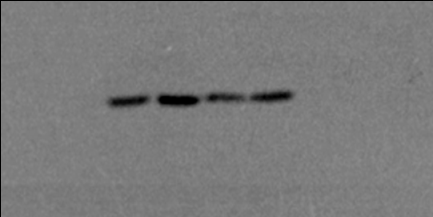
**

**Vimentin**

**
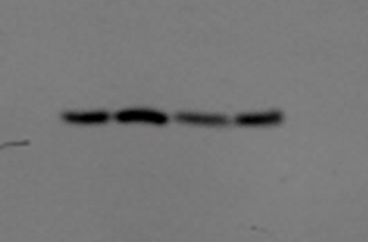
**

**Figure 6G-BT-549 cell**

**GAPDH**

**
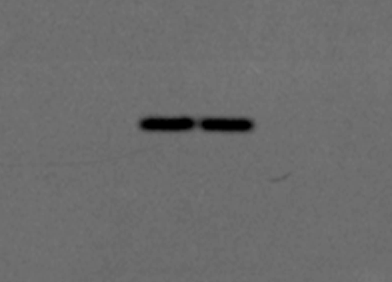
**

**SOCS2**

**
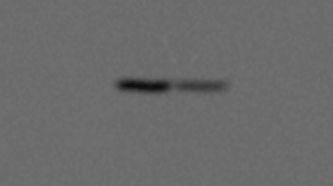
**

**Figure 6G-MDA-MB-231 cell**

**GAPDH**

**
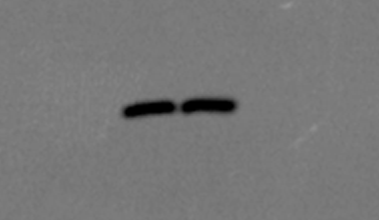
**

**SOCS2**

**
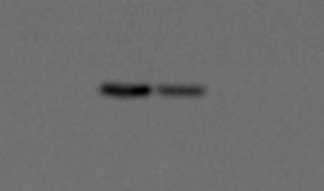
**

**Figure 6H-BT-549 cell**

**GAPDH**

**
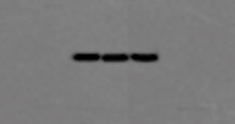
**

**SOCS2**

**
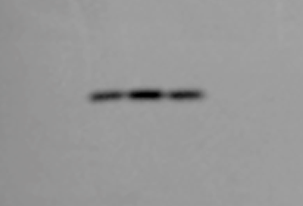
**

**p-STAT5**

**
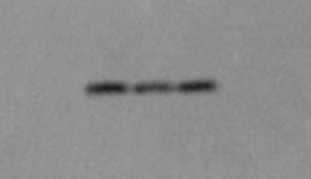
**

**STAT5**

**
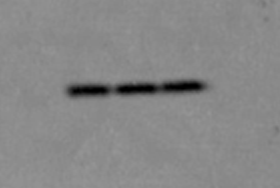
**

**p-JAK2**

**
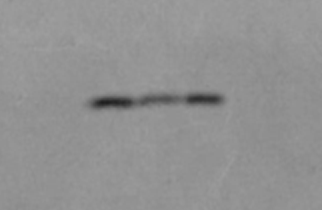
**

**JAK2**

**
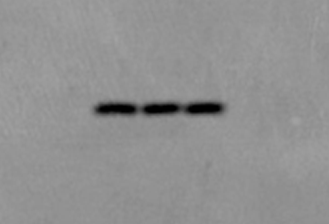
**

**Figure 6H-MDA-MB-231 cell**

**GAPDH**

**
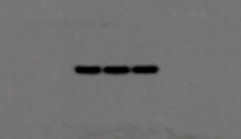
**

**SOCS2**

**
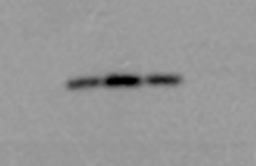
**

**p-STAT5**

**
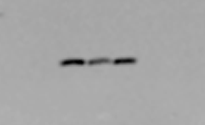
**

**STAT5**

**
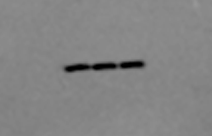
**

**p-JAK2**

**
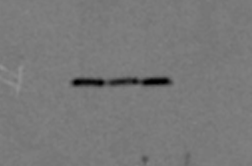
**

**JAK2**

**
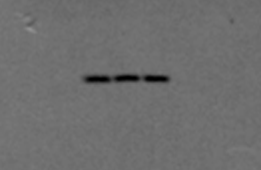
**

**Figure 7A-BT-549 cell**

**GAPDH**

**
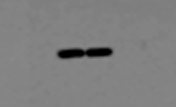
**

**SOCS2**

**
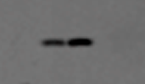
**

**Figure 7A-MDA-MB-231 cell**

**GAPDH**

**
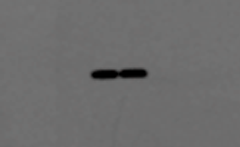
**

**SOCS2**

**
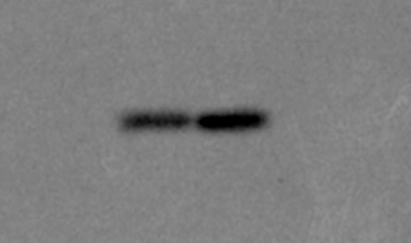
**

**Figure 7F-BT-549 cell**

**GAPDH**

**
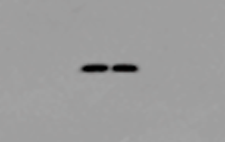
**

**E-cadherin**

**
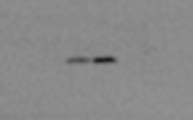
**

**N-cadherin**

**
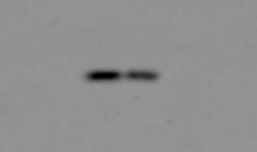
**

**Vimentin**

**
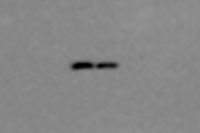
**

**Figure 7F-MDA-MB-231 cell**

**GAPDH**

**
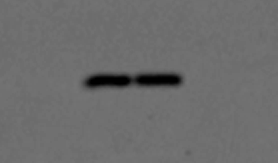
**

**E-cadherin**

**
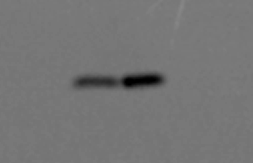
**

**N-cadherin**

**
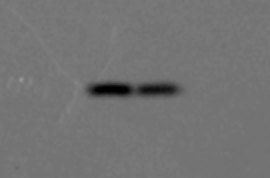
**

**Vimentin**

**
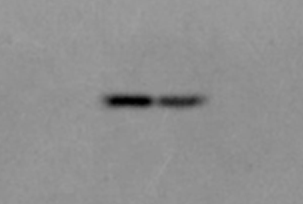
**

**Figure 9E**

**GAPDH**

**
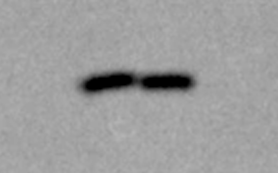
**

**SOCS2**

**
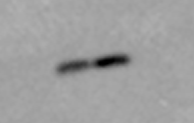
**

**E-cadherin**

**
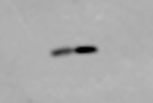
**

**N-cadherin**

**
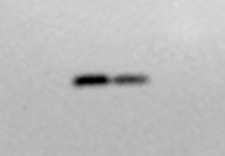
**
